# Caloric and Macronutrient Intake Differ with Circadian Phase and between Lean and Overweight Young Adults

**DOI:** 10.3390/nu11030587

**Published:** 2019-03-11

**Authors:** Andrew W. McHill, Charles A. Czeisler, Andrew J. K. Phillips, Leigh Keating, Laura K. Barger, Marta Garaulet, Frank A. J. L. Scheer, Elizabeth B. Klerman

**Affiliations:** 1 Division of Sleep and Circadian Disorders, Departments of Medicine and Neurology, Brigham and Women’s Hospital, 221 Longwood Ave, Boston, MA 02115, USA; caczeisler@reserach.bwh.harvard.edu (C.A.C.); andrew.phillips@monash.edu (A.J.K.P.); laura_barger@hms.harvard.edu (L.K.B.); fscheer@bwh.harvard.edu (F.A.J.L.S.); ebklerman@research.bwh.harvard.edu (E.B.K.); 2 Division of Sleep Medicine, Department of Medicine, Harvard Medical School, 221 Longwood Ave, Boston, MA 02115, USA; 3 Oregon Institute of Occupational Health Sciences, Oregon Health & Science University, 3181 SW Sam Jackson Park Road, Portland, OR 97239, USA; 4 Monash Institute of Cognitive and Clinical Neurosciences, School of Psychological Sciences, Monash University, 18 Innovation Walk, Clayton, VIC 3800, Australia; 5 Center for Clinical Investigation, Brigham and Women’s Hospital, 75 Francis Street, Boston, MA 02115, USA; leigh.keating@childrens.harvard.edu; 6 Department of Physiology, University of Murcia and Research Biomedical Institute of Murcia (IMIB), 30100 Murcia, Spain; garaulet@um.es

**Keywords:** metabolism, sleep duration, body composition, caloric intake, melatonin

## Abstract

The timing of caloric intake is a risk factor for excess weight and disease. Growing evidence suggests, however, that the impact of caloric consumption on metabolic health depends on its circadian phase, not clock hour. The objective of the current study was to identify how individuals consume calories and macronutrients relative to circadian phase in real-world settings. Young adults (*n* = 106; aged 19 ± 1 years; 45 females) photographically recorded the timing and content of all calories for seven consecutive days using a smartphone application during a 30-day study. Circadian phase was determined from in-laboratory assessment of dim-light melatonin onset (DLMO). Meals were assigned a circadian phase relative to each participant’s DLMO (0°, ~23:17 h) and binned into 60° bins. Lean (*n* = 68; 15 females) and non-lean (*n* = 38, 30 females) body composition was determined via bioelectrical impedance. The DLMO time range was ~10 h, allowing separation of clock time and circadian phase. Eating occurred at all circadian phases, with significant circadian rhythmicity (*p* < 0.0001) and highest caloric intake at ~300° (~1900 h). The non-lean group ate 8% more of their daily calories at an evening circadian phase (300°) than the lean group (*p* = 0.007). Consumption of carbohydrates and proteins followed circadian patterns (*p* < 0.0001) and non-lean participants ate 13% more carbohydrates at 240° (~1500 h) than the lean group (*p* = 0.004). There were no significant differences when caloric intake was referenced to local clock time or sleep onset time (*p* > 0.05). Interventions targeting the circadian timing of calories and macronutrients for weight management should be tested.

## 1. Introduction

Excess body weight and obesity are common in industrialized societies. According to the National Health and Nutrition Examination Survey conducted in 2013–2014, approximately 70% of the US adult population was classified as overweight and/or obese, with approximately 40% of that population falling into the obese category [1]. In Europe, over 50% of the adult European Union is classified as being overweight [2]. Overweight and obese body weight is associated with an increased risk for heart disease, stroke, diabetes, and cancer [3] and accounts for ~147 billion dollars in health care costs in the United States each year [4]. However, despite the known consequences of excess body weight, recent trends have suggested that the percentages of overweight and obese individuals continue to rise worldwide [5]. Thus, identifying potential modifiable behaviors that could decrease the continued rise of excess body weight is vital to combatting disease.

The timing of daily food consumption is a novel risk factor for higher body fat percentage and disease [6]. Recently, researchers using a mobile phone food tracking application found that many individuals in free-living settings lack a traditional 3-meal-a-day pattern, and consume calories erratically at all times of the day and night [7,8]. These studies, however, reference caloric intake to local 24 h clock time, not the more physiological timing of the phase of each individual’s endogenous circadian clock. We have previously shown that the time at which an individual consumes 50% of their daily caloric intake (caloric midpoint) relative to the individual’s endogenous circadian phase may play a more important role in body composition than local clock timing of their intake [9]. Furthermore, recent in-laboratory data demonstrate that human energy expenditure and macronutrient oxidation differ depending on circadian phase [10]. This raises the question of whether calories eaten at various times of day may differentially influence energy balance. Indeed, energy expenditure following an identical test meal (diet-induced thermogenesis) is substantially lower in the circadian evening as compared to the circadian morning [11,12]. Thus, the specific circadian phase of food intake in real-world settings could play a role in excess body weight if eating predominantly occurs during the circadian evening. This may be of additional importance when considering the timing of food consumption in relation to the timing of sleep. Indeed, when examining this relationship in cohort studies, individuals who consume a larger portion of their calories, particularly carbohydrates and proteins, close to their habitual bedtime—which is a better proxy measure of circadian phase than clock time—have higher odds of having an overweight or obese body mass index (BMI), while no relationship was shown when using clock time [13]. Therefore, identifying potential relationships between patterns of caloric intake and timing of sleep onset could lead to improved weight management strategies. Finally, previous reports that associate short sleep with weight gain are confounded by the fact that participants in short sleep conditions eat a higher proportion of their daily calories during the night time hours [14,15,16].

In the current cross-sectional study, we examined the circadian distribution of percentage of daily caloric and macronutrient intake in free-living settings using a photographic mobile phone application across a 7-day meal-tracking protocol. Specifically, we wanted to examine the circadian profiles of caloric and macronutrient intake in an individual’s habitual real-world setting. We hypothesized that caloric consumption would follow a circadian pattern and that non-lean individuals would eat a higher percentage of their daily calories with non-uniform distribution of fat, carbohydrates, and protein at a later circadian phase as compared to lean individuals. As a secondary analysis, we also wanted to analyze timing of calorie consumption relative to timing of sleep.

## 2. Materials and Methods

### 2.1. Participants

Participants (*n* = 106, 45 females) aged 19 ± 1, 18–22 years (mean ± standard deviation, range) with an average BMI of 23.0 ± 3.8, 16.2–42.8 kg/m^2^ (Supplementary Table S1) were recruited from a local Boston university using paper flyers around campus, email, and verbal communication. Inclusion criteria consisted of the ability to download the food tracking mobile phone application, ability to wear an actigraphy monitor, no current night-work during the protocol, and no travel of more than one time zone in the three months prior to and throughout the protocol. There were no other exclusion criteria. All participants provided written informed consent prior to any data collection and all study procedures were approved by the Partner’s Healthcare Institutional Review Board. This study was registered at clinicaltrials.gov (NCT02846077).

### 2.2. Field-Study Procedures

Upon learning detailed information about the protocol from study staff, participants volunteered to be enrolled in an approximately 30-day protocol to record sleep, food intake patterns, and circadian phase within their habitual routines [9,17].

For the 30 days of the study, participants wore a wrist actigraphy monitor (MotionLogger; Ambulatory Monitoring, Ardsley, NY, USA) on their non-dominant arm at all times except for when the monitor might get wet or damaged, and completed electronic sleep-wake and exercise diaries once each morning (~07:00 h) and once each evening (~20:00 h).

For seven consecutive days during the 30 days of monitoring, participants recorded all food and beverages they consumed using the photographic mobile phone application MealLogger^TM^ (Wellness Foundry, New York, NY, USA) that time-stamped the clock-time of their meal and enabled participants to leave a detailed description of the meal content (e.g., any type of salad dressing or condiments used or additives such as milk or sugar to beverages) [9]. Upon completion of each caloric entry, data were available to study staff via web access and nutrition staff followed up with participants through the mobile app within 24 h after the meal was documented if any clarification of meal composition was needed. Participants were instructed to include an object of known size within the picture to help calculate portion size and to take a second photo if the meal was not fully consumed to estimate total caloric intake. If a participant recorded ≤2 caloric events within a waking day, study staff emailed them to confirm they had not consumed any additional calories.

### 2.3. In-laboratory Procedures

Once during the 30 days, participants were admitted to the Brigham and Women’s Hospital Center for Clinical Investigation Intensive Physiologic Monitoring Unit for an approximately 16 h overnight stay to assess body composition and dim-light melatonin onset (DLMO) timing as a measure of circadian phase. This in-laboratory visit occurred within an average of 7.2 days (median 3 days, range 12 days before to 22 days after) from the 7-day food diary collection in order to minimize the impact of sleep loss from the in-laboratory visit on subsequent eating patterns. Upon arrival to the laboratory, participants removed jewelry and wrist-worn devices and lay supine on a bed; research staff placed electrodes on the participant’s right hand and foot to measure body composition using a four-lead bioelectrical impedance device (Quantum II BIA analyzer, RJL Systems, Clinton Township, MI, USA). Each impedance measurement was performed three times to confirm consistent results and an average of these three readings was used for analysis. Each participant was provided a pre-selected evening meal of standard size (e.g., sandwich and salad) and supplemental snacks if requested (i.e., potato chips), but were not required to eat the meal or snacks in their entirety. They were also provided a standardized snack upon exiting the laboratory. No food was provided that would directly affect sleep (e.g., caffeine) or melatonin rhythms (e.g., bananas). Note that none of the dietary data during the laboratory visit was used for meal timing analysis.

Salivary melatonin samples were collected hourly in dim-light conditions (<4 lux) beginning at ~16:00 h and ending at ~07:00 h the next morning (a total of 16 samples per subject). To minimize any potential exogenous influences on melatonin concentrations, participants were not allowed to use any personal light-emitting electronic devices while in the laboratory. Additionally, for 20 min immediately prior to each saliva sample collection, participants were instructed to refrain from eating or drinking and to maintain a constant seated posture. For the next 40 min within each hour, participants were allowed to remain seated, eat a provided snack, ambulate within the dim study room, or sleep in a seated position. If participants chose to sleep, they were awakened by research staff immediately prior to saliva collection.

### 2.4. Analysis

Actigraphic sleep timing and duration were manually scored using sleep onset and offset times from the electronic diary sleep-wake entries [9,18]. Food intake entries were assessed for caloric content and macronutrient composition independently by two research dieticians within the Brigham and Women’s Hospital Center for Clinical Investigation using the University of Minnesota Nutrition Data System for Research software [19,20]. Caloric entries that were consumed within 15 minutes of each other and identified by the participant as the same type of meal (e.g., lunch) were combined into one ‘caloric event’ [7,9]. Participants with <4 calendar days of meal tracking were excluded from analysis.

Circadian phase was determined for each participant using the DLMO, defined as the linear interpolated point in time at which melatonin levels crossed and maintained concentrations above a 5 pg/mL threshold [21]. Each caloric event entered by the participant was assigned a circadian phase relative to the timing of that participant’s DLMO (0°, 23:17 h on average in our study population) Data were binned into 60° bins (15° denotes 1 h, thus 4 h bins) per day. When examining percentage of daily caloric intake, if a participant did not have a caloric event during a specific circadian bin (e.g., 120°), that bin was assigned a zero-caloric value prior to averaging bins across the week for that participant. For measurement of macronutrient intake, only bins that contained caloric intake were used for analysis. The percentage of time each individual was awake for each circadian bin during the week of meal monitoring was also calculated using the actigraphy and daily diary information. Caloric events were also binned by local clock time and time relative to actigraphically scored sleep onset (4 h bins).

Participants were classified as having a lean or non-lean body composition using sex-dependent criteria for percent body fat [22]. Lean individuals were defined as having a percent body fat <31% for females and <21% for males; the non-lean group was defined as ≥31% body fat for females and ≥21% for males [22].

Descriptive characteristics between the lean and non-lean group were analyzed using unpaired t-tests. Caloric and macronutrient intake across circadian phases, local clock hour, and time relative to sleep onset were analyzed using mixed-effects models (variance components) with circadian phase, clock hour, or time relative to sleep onset as a categorical fixed factor, participant as a random factor to account for inter-participant differences, and sex as a covariate. Caloric and macronutrient intake between body composition groups and across circadian phase, clock hour, and time relative to sleep onset bins were analyzed with circadian phase, clock hour, or time relative to sleep onset and group (lean vs. non-lean with sex-specific criteria) as fixed factors, participant as a random factor, and sex as a covariate. Planned post-hoc comparisons were performed for differences between groups at each time point with t-tests applying a Bonferroni correction (*p* < 0.008 needed to reach significance) to account for multiple comparisons. All statistical analyses were performed using SAS 9.4 (SAS Institute Inc., Cary, NC, USA).

## 3. Results

### 3.1. Circadian and Local Timing

DLMO ranged from 17:52 to 03:38 h (local clock time) and sleep onset timing and duration during the 7 days of meal tracking ranged from 23:59 to 05:17 h and 5.5 to 9.3 h, respectively (Supplementary Table S1). The difference between sleep onset time and DLMO ranged from −0.41 to 8.30 h with a mean of 3.35 h (SD 1.40 h).

### 3.2. Caloric Intake Across Circadian Phases and Local Clock Time

**Figure 1:**
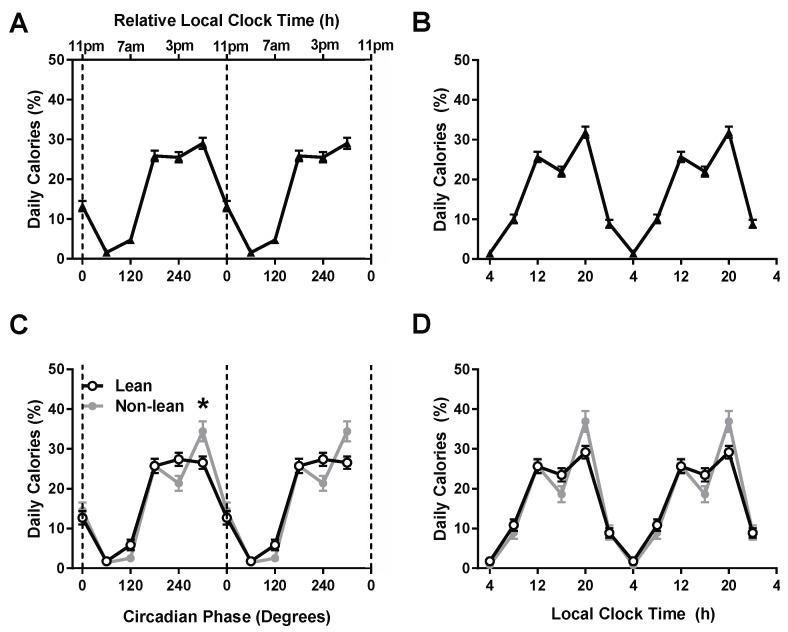
Caloric intake across circadian phases and local clock time. Influence of circadian and local clock timing on percentage of daily calories in all participants (**A**,**B**) and between lean and non-lean participants (**C**,**D**). All participants (*n* = 106) are denoted as triangles, the lean group (*n* = 68) is denoted by open circles and the non-lean group (*n* = 38) by closed gray circles. Data are double plotted across circadian phase (0° denotes timing of dim-light melatonin onset) and relative local clock time based on the group average dim-light melatonin of ~23:00 or across local clock time. The * symbol denotes a significant difference after Bonferroni correction (*p* = 0.007) between lean and non-lean groups. Error bars represent standard error of the mean.

Across all participants, eating occurred in all circadian phase bins, with a significant circadian phase effect (F_5,1722_=14.5, *p* < 0.0001) exhibiting a nadir of caloric consumption during the biological night in the bin centered at 60° (equivalent to ~03:00 h on average) and a peak during the biological evening in the bin centered at 300° (~19:00 h; Figure 1A). When analyzing caloric intake according to local clock time, eating also occurred in all clock time bins, with a significant clock time effect (F_5,1722_ = 14.74, *p* < 0.0001) with calories peaking at ~20:00 h (Figure 1B). Next, we examined differences in the temporal pattern of caloric consumption between the lean and non-lean groups. There was a significant circadian phase–by-group interaction (F_5,1717_ = 2.63, *p* =0.02) and a significant circadian phase effect (F_5,1717_ = 16.27, *p* < 0.0001), but no group effect (F_1,1717_ = 1.47, *p* = 0.22) (Figure 1C). Lastly, we performed planned post-hoc analyses between groups at each time bin with t-tests applying a Bonferroni correction (*p* < 0.008 needed to reach significance) to account for multiple comparisons. The non-lean group ate 8% more of their daily calories during the biological evening at 300° (~19:00 h) as compared to the lean group (t(104)= −2.72, *p* = 0.007), without significant differences for the other circadian bins (all *p* > 0.03) (Figure 1C). Notably, when analyzing the data according to local clock time, and in contrast to the analysis with respect to circadian phase, there were no significant group or group-by-local clock time interactions between lean and non-lean groups (all *p* > 0.25; Figure 1D), including post-hoc analyses at any local clock time (all *p* > 0.05).

### 3.3. Macronutrient Intake Across Circadian Phases and Local Time

**Figure 2:**
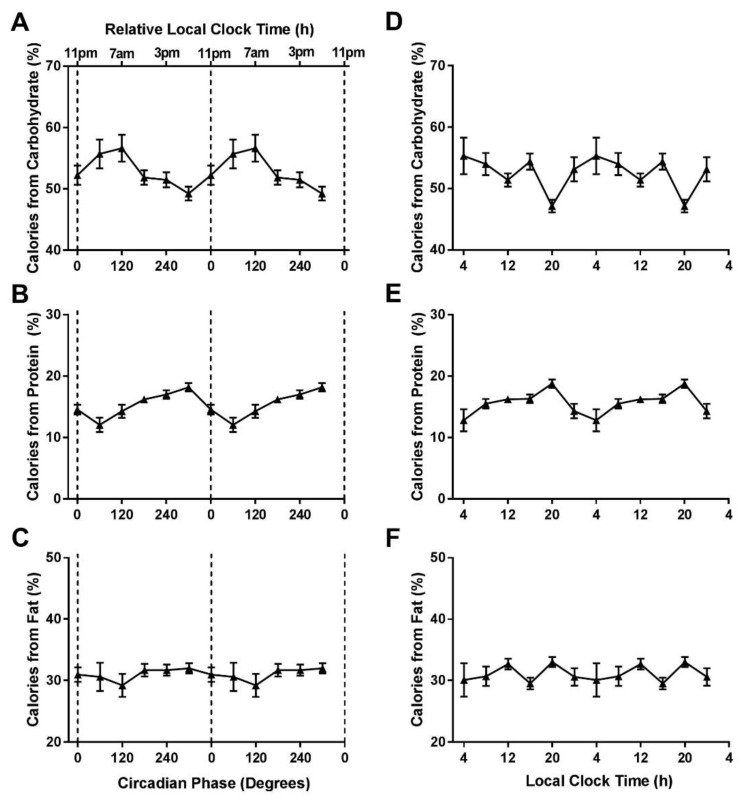
Macronutrient intake across circadian phases and local time. Influence of circadian phase (**A**–**C**) and local clock timing (**D**–**F**) on percentage of daily calories from carbohydrates, proteins, and fats. Data are double plotted across circadian phase (0° denotes timing of dim-light melatonin onset) and relative local clock time based on the group average dim-light melatonin of ~23:00. Error bars represent standard error of the mean.

There were significant circadian phase effects for percentage of calories from carbohydrates (F_5,1728_ = 3.30, *p* = 0.006) and proteins (F_5,1728_ = 7.17, *p* < 0.0001), with carbohydrates and proteins peaking at 120° and 300° and a nadir at 300° and 60°, respectively (Figure 2A,B). There was no significant circadian phase effect for the percentage of calories from fat (F_5,1728_ = 1.20, *p* = 0.31, Figure 2C). There were significant local clock time effects for carbohydrates (F_5,1728_ = 8.61, *p* < 0.0001), proteins (F_5,1728_ = 8.54, *p* < 0.0001), and fats (F_5,1728_ = 2.91, *p* = 0.01), with peaks occurring at ~20:00 h for proteins and fats and ~04:00 h for carbohydrates (Figure 2D–F).

**Figure 3:**
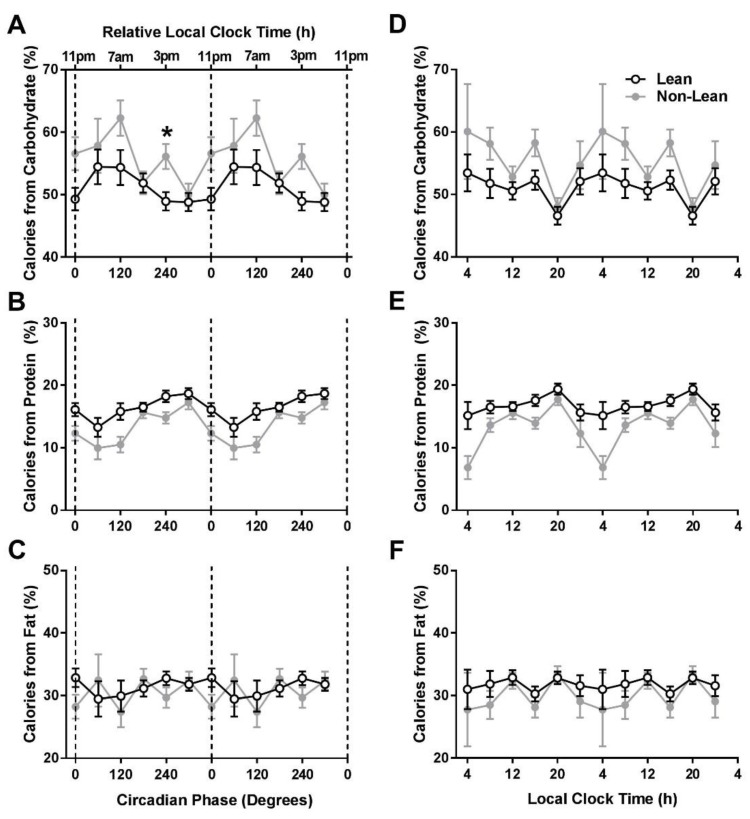
Macronutrient intake across circadian phases and local time in lean and non-lean individuals. Influence of circadian phase (**A**–**C**) and local clock timing (**D**–**F**) on percentage of daily calories from carbohydrates, proteins, and fats between lean and non-lean individuals. The lean group (*n* = 68) is denoted by open circles and the non-lean group (*n* = 38) by closed gray circles. Data are double plotted across circadian phase (0° denotes timing of dim-light melatonin onset) and relative local clock time based on the group average dim-light melatonin of ~23:00 or across local clock time. The * symbol denotes a significant difference after Bonferroni correction (*p* = 0.004) between lean and non-lean groups. Error bars represent standard error of the mean.

When comparing the lean vs. non-lean groups’ macronutrient intake across circadian phases, for percentage of calories from carbohydrates, there was a significant circadian phase-by-group interaction (F_5,1723_ = 2.27, *p* = 0.04) and a significant circadian phase effect (F_5,1723_ = 3.66, *p* = 0.003). For percentage of calories from proteins, there were no significant circadian phase–by-group interaction (F_5,1723_ = 1.60, *p* = 0.16) or group effects (F_1,1723_ = 2.54, *p* = 0.11). However, there was a significant circadian phase effect (F_5,1723_ = 8.02, *p* < 0.0001; Figure 3A–C). For percentage of calories from fat, there was no significant group effect, circadian phase effect, or circadian phase–by-group interaction (all *p* > 0.16). In post-hoc analyses, the non-lean participants ate a higher percentage of calories from carbohydrates at 240° as compared to the lean group (t(100) = −2.93, *p* = 0.004).

When analyzing macronutrient intake based on local clock time, for percentage of calories from carbohydrates, proteins, and fat, there were no significant time of day-by-group interactions or lean vs. non-lean group effects (all *p* > 0.05; Figure 3D–F).

### 3.4. Calories Relative to Time of Sleep Onset

We next examined whether changes in the percentage of calories consumed across circadian phases was influenced by the timing of sleep. There was a significant circadian phase effect for percentage of time awake (Supplementary Figure S1A, F_5,485_ = 301.9, *p* < 0.0001) and a significant local clock time effect for percentage of time awake (Supplementary Figure S1B, F_5,490_ = 447.3, *p* < 0.0001). For group comparisons of percentage of time awake, there was a significant circadian phase–by-group interaction (F_5,480_ = 3.74, *p* = 0.003) circadian phase effect (F_5,480_ = 288.51, *p* < 0.0001) (Supplementary Figure S1C), and local clock time effect (F_5,485_ = 421.36, *p* < 0.0001), but no local clock time-by-group interaction (F_5,485_ = 1.15, *p* = 0.33; Supplementary Figure S1).

To understand the relationship between caloric events and sleep timing, we examined percentage of calories and macronutrients consumed relative to the timing of sleep onset. The percentage of calories consumed and percentage of calories from carbohydrates, proteins, and fats differed depending on time before sleep onset (all *p* < 0.05; Supplementary Figure S2A–D). There were no significant group-by-time from sleep onset interactions or group effects for differences in daily calories and percentage of calories from carbohydrates, fats, and proteins (all *p* > 0.08) (Supplementary Figure S2E–H).

## 4. Discussion

With the widespread use of electrical lighting in modern societies, humans have the ability to extend work and social activities across all times of the 24 h day [23,24], which also enables individuals to consume calories at all times of the 24 h day. In the current study, we showed for the first time that caloric intake occurs across all phases of the circadian clock, and that non-lean individuals tend to eat a larger percentage of their daily calories at a later circadian phase than lean individuals. Importantly, these differences between body composition groups were only present when aligned with a physiological marker (circadian phase), and not local clock hour or a behavioral marker (time from sleep onset). These findings provide valuable insight into a potentially modifiable behavior, the circadian timing of caloric intake, which can be targeted in future interventions to reduce weight gain and comorbid disease.

Our findings that the percentage of calories consumed differs depending on circadian phase and local clock hour aligns closely with previous studies examining the diurnal patterns of caloric consumption. Using paper food diaries to track the clock hour and content of foods consumed, de Castro and colleagues found that the peak time of caloric consumption occurred at approximately 19:00 h [25] and using a mobile phone food tracking application, Gupta and colleagues found a peak in eating events at approximately 20:00 h [8]. Our data demonstrate a similar peak time in percentage of daily calories (~19:00 h); however, we now report where these calories occur relative to circadian phase (at 300°). One reason for the difference (clock vs. circadian time) may be the wide (~10 h) range in circadian phases in this population. Interestingly, our caloric intake data from young adults are in agreement with the circadian peak timing of subjective hunger from tightly-controlled in-laboratory studies [26,27], suggesting that the observed subjective circadian rhythm of hunger may contribute importantly to actual intake in real-world settings. Examining caloric consumptions simultaneously with subjective hunger in laboratory studies and in habitual settings are needed to fully understand this relationship. Moreover, these data need to be replicated in different parts of the world to begin to separate potential cultural influences on the temporal distribution of caloric and macronutrient intake.

The potential for the timing of calories to influence body composition has been previously described in the literature. However, differences in the circadian phase of these calories and macronutrients in real-world settings have not been documented. Baron and colleagues found that calories consumed after 20:00 h are associated with a higher BMI when controlling for sleep timing and duration [28], and that higher amounts of macronutrients after 20:00 h was also associated with higher BMI [29]. Consistently, those that eat higher percentages of their calories earlier in the day have greater effectiveness in attempted weight loss [30,31,32]. Restricting calories to daytime hours, with no restrictions on caloric intake or meal composition, has also been found to reduce body weight [7] and improve other cardiometabolic markers [33]. Moving calories earlier in the day may also reduce the total amounts of calories consumed [34,35,36], which in turn would promote weight loss. Although these types of interventions have resulted in positive health benefits, protocols tailoring interventions to include internal circadian timing may have larger effects. Within the current study, our similarly-aged individuals living in the same city, exhibited an ~10 h inter-individual difference in the timing of DLMO. Thus, a strict cut-off time to stop consuming calories, such as 20:00 h, may work well for some individuals, but not as well for others. This point is of further importance in the context of aging, since the timing of DLMO, sleep onset, and the subsequent difference between DLMO and sleep onset, change with age [37,38]. Randomized trials are needed to test differences in weight loss and other outcomes when using circadian timing as opposed to clock hour of restricted feeding.

We also examined the macronutrient composition of calories consumed. Interestingly, we found significant circadian and group interaction effects for the percentage of carbohydrates consumed, but not for percentage of fats and proteins. Previously, the consumption of fats, carbohydrates, and proteins have been found to follow a similar diurnal pattern to overall caloric intake, with bimodal peaks at around noon and 19:00 h [25]. This is also true for the circadian rhythm in desire for starchy and meats/poultry types of food, with a peak during the circadian evening [26]. We found that our population ate foods higher in carbohydrates earlier in the day and higher protein foods later in the day, however the physiological importance of the differing patterns observed in macronutrient intake between the lean and non-lean individuals is not clear. There is evidence that carbohydrate oxidation is decreased at later circadian phases [10,11,39,40,41] and that increased protein diets may lower appetite and subsequent caloric intake [42]. Interestingly, the circadian rhythm of carbohydrate oxidation mirrors our findings of carbohydrate intake, with a nadir in the evening hours [10]. How these mechanisms may alter body composition in regards to daily caloric intake in our population is unknown. Future work is needed to examine the direct impact of providing differing macronutrient compositions and their subsequent oxidations at differing circadian phases to directly test this impact.

To help understand if changes in calories consumed across circadian phases was influenced by the timing of sleep, we examined percentage of time awake across circadian phases and local clock time. In doing so, we found that participants were likely to be awake and eat across all circadian phases and times of day, and that there was no difference in caloric intake between lean and non-lean groups relative to sleep onset. Previous work has shown that individuals that consume a higher percentage of their calories closer to their habitual bedtime have higher odds of being overweight or obese [13]. As that was a much larger cohort study (*n* = 872), our current findings may not have been powered to find a difference in calories consumed relative to sleep onset and only caloric intake relative to the more precise circadian phase assessed by DLMO (for which we found a significant difference). However, the relationship between caloric intake and circadian phase may be of importance because energy expenditure follows a circadian pattern, with lowest levels during the circadian night [10], and circadian evening caloric intake may be coupled with a decreased diet-induced thermogenesis [11,12,43]. This is supported by observational findings that have shown that individuals with a later circadian phase have a lower BMI [44], potentially due to increasing the time interval between meals and the circadian night if the clock time of meal intake were to stay consistent between those with earlier circadian phase.

Our study has several limitations to consider when interpreting the findings. The nature of the cross-sectional study design in real-world settings is not ideal for determining the circadian timing of events, as the timing of sleep is most likely to occur at certain circadian phases [45] and will have an impact on the ability to consume calories. Use of a forced desynchrony protocol [46], where events are scheduled to occur evenly across all circadian phases, as well as access to ad libitum food intake, would be needed to fully elucidate a circadian rhythm to caloric consumption. To account for timing of sleep, we examined the percentage of daily calories consumed per time awake and did observe that individuals in our group ate and slept at all circadian phases and across all local clock times of the day. Thus, we hypothesize that our results accurately reflect the relationship between circadian timing and caloric consumption in real-world settings in this population. Further, our study population of young adults may not accurately reflect the eating patterns and composition of calories consumed by other populations. By matching the timing of caloric events to each individual’s DLMO, however, we were able to account for each individual’s circadian pattern of caloric intake, which we hypothesize would be similar for other populations not working overnight shiftwork. Lastly, due to the study design, we were only able to collect one measure of DLMO across the 30-day protocol; this measure, however, was within ~7 days on average of the 7-day food diary collection. Although this may limit our ability to match small changes in daily DLMO with eating patterns, due to the size of our data binning (~4 h) and the magnitude of the daily shift induced by typical room lighting [47,48], we do not predict this will drastically impact our results or conclusions.

## 5. Conclusions

In summary, our findings suggest that the timing of caloric consumption differs depending on circadian phase in real-world settings, with non-lean individuals eating a greater percentage of calories at a later circadian phase. These findings potentially highlight a therapeutic area to target to combat the rise in unhealthy body composition. Further, these data reflect the importance of considering each individual’s circadian timing, and not just clock time, when devising therapeutic strategies that combat the timing of caloric intake.

## Supplementary Materials

The following are available online at https://www.mdpi.com/2072-6643/11/3/587/s1, Supplementary Table S1: Participant characteristics, Sleep and Circadian Timing; Supplemental Figure S1: Percent of Time Awake across Circadian Phases and Local Time.; Supplemental Figure S2: Calories Relative to Time of Sleep Onset.
