# Supplementary material for: Caloric and Macronutrient Intake Differ with Circadian Phase and between Lean and Overweight Young Adults
Source: Nutrients. 2019 Mar 11;11(3):587. doi: 10.3390/nu11030587 (PMC6471585; doi:10.3390/nu11030587)
Supplement: Supplementary file 1 [file nutrients-11-00587-s001.pdf]

**Supplemental Table S1**. Participant characteristics, Sleep and Circadian Timing.

| **Measure** | **All** (n = 106) | **Lean** (n = 68) | **Non-lean** (n = 38) | **p-value** |
|---|---|---|---|---|
| *Sex* | | | | |
| Male, n(%) | 61 (57.5) | 53 (77.9) | 8 (21.1) | N/A |
| Female, n(%) | 45 (42.5) | 15 (22.1) | 30 (78.9) | N/A |
| *Body Composition and Activity* | | | | |
| BMI (kg/m2) | 23.0 (3.8) | 21.8 (2.6) | 25.1 (4.7) | p< 0.001 |
| Body Fat Percentage (%) | 22.5 (9.5) | 17.0 (5.9) | 32.3 (6.5) | p< 0.001 |
| *Days with Self-reported Exercise (%)* | 26.3 (24.3) | 27.0 (25.2) | 25.0 (22.9) | p = 0.68 |
| *Duration of Self-reported Exercise (h/d)* | 1.2 (0.6) | 1.2 (0.5) | 1.3 (0.7) | p = 0.43 |
| *Dietary Intake* | | | | |
| Daily Average Energy Intake (kJ) | 6950 (2000) | 6998 (2046) | 6863 (1938) | p = 0.74 |
| Daily Average Fats (% Total Energy) | 34.1 (4.5) | 34.0 (4.5) | 34.4 (4.7) | p = 0.61 |
| Daily Average Carbohydrates (% Total Energy) | 47.3 (5.9) | 46.8 (6.0) | 48.1 (5.6) | p = 0.30 |
| Daily Average Proteins (% Total Energy) | 18.1 (4.0) | 18.6 (4.0) | 17.0 (3.7) | p = 0.04 |
| *Circadian and Sleep Measures* | | | | |
| Circadian Timing (Dim-Light Melatonin Onset) (hh:min) | 23:17 (01:41) | 23:25 (01:47) | 23:03 (01:30) | p = 0.29 |
| Sleep Onset (hh:min) | 02:37 (01:07) | 02:34 (01:11) | 02:41 (01:00) | p = 0.61 |
| 24h Total Sleep Time (h) | 7.0 (0.7) | 7.1 (0.8) | 7.0 (0.6) | p = 0.64 |

Values are means (SD) unless otherwise specified. P-values represent comparisons between lean and non-lean values. Note; due to large differences in regards to the percentage of males and females characterized as lean or non-lean, sex was included as a covariate in all analysis. Days and durations of the self-reported exercise were calculated for the entire 30-day protocol within each individual and then averaged across individuals.

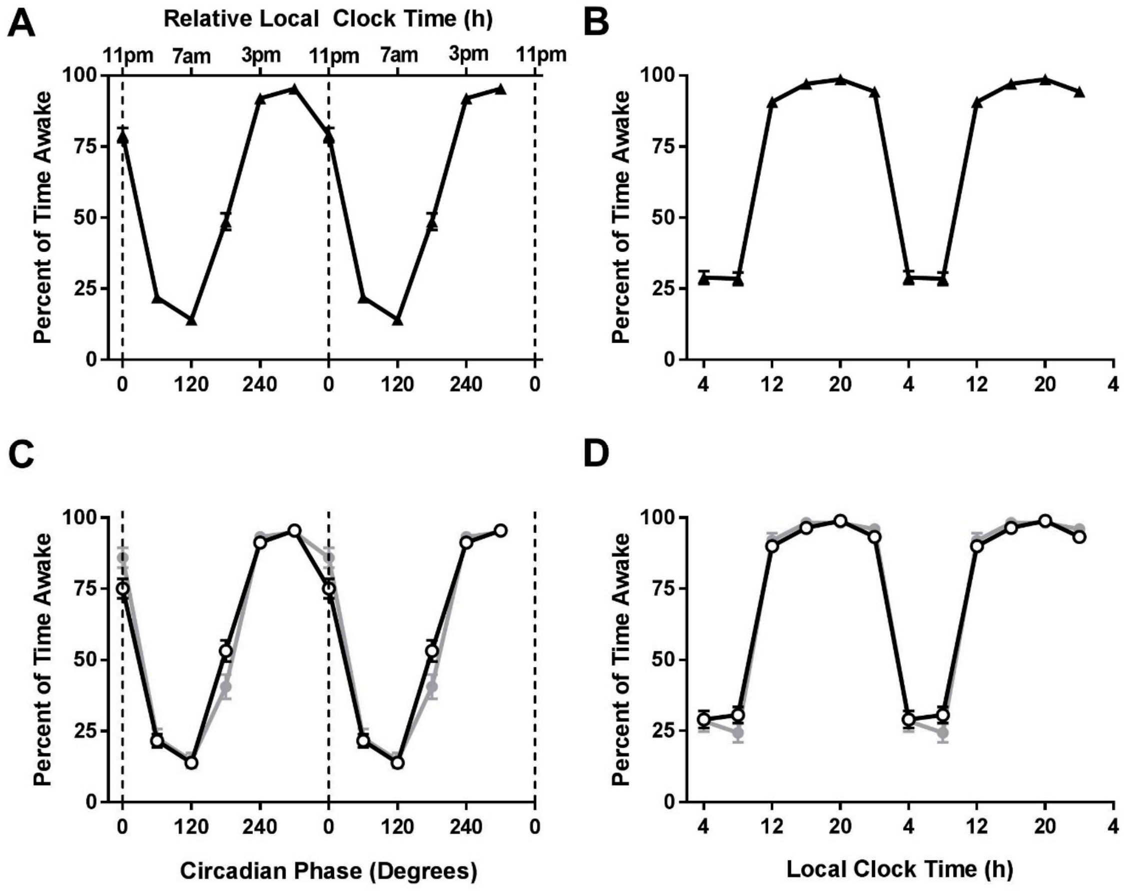


**Supplemental Figure S1.** Percent of Time Awake Across Circadian Phases and Local Time. Influence of circadian and local clock timing on percent of time awake in all participants (A, B) and between lean and non-lean participants (C, D). All participants (n = 106) are denoted as triangles, the lean group (n = 68) is denoted by open circles and the non-lean group (n = 38) by closed gray circles. Data are double plotted across circadian phase (0° denotes timing of dim-light melatonin onset) and relative local clock time based on the group average dim-light melatonin of ~23:00 or across local clock time. Error bars represent standard error of the mean.

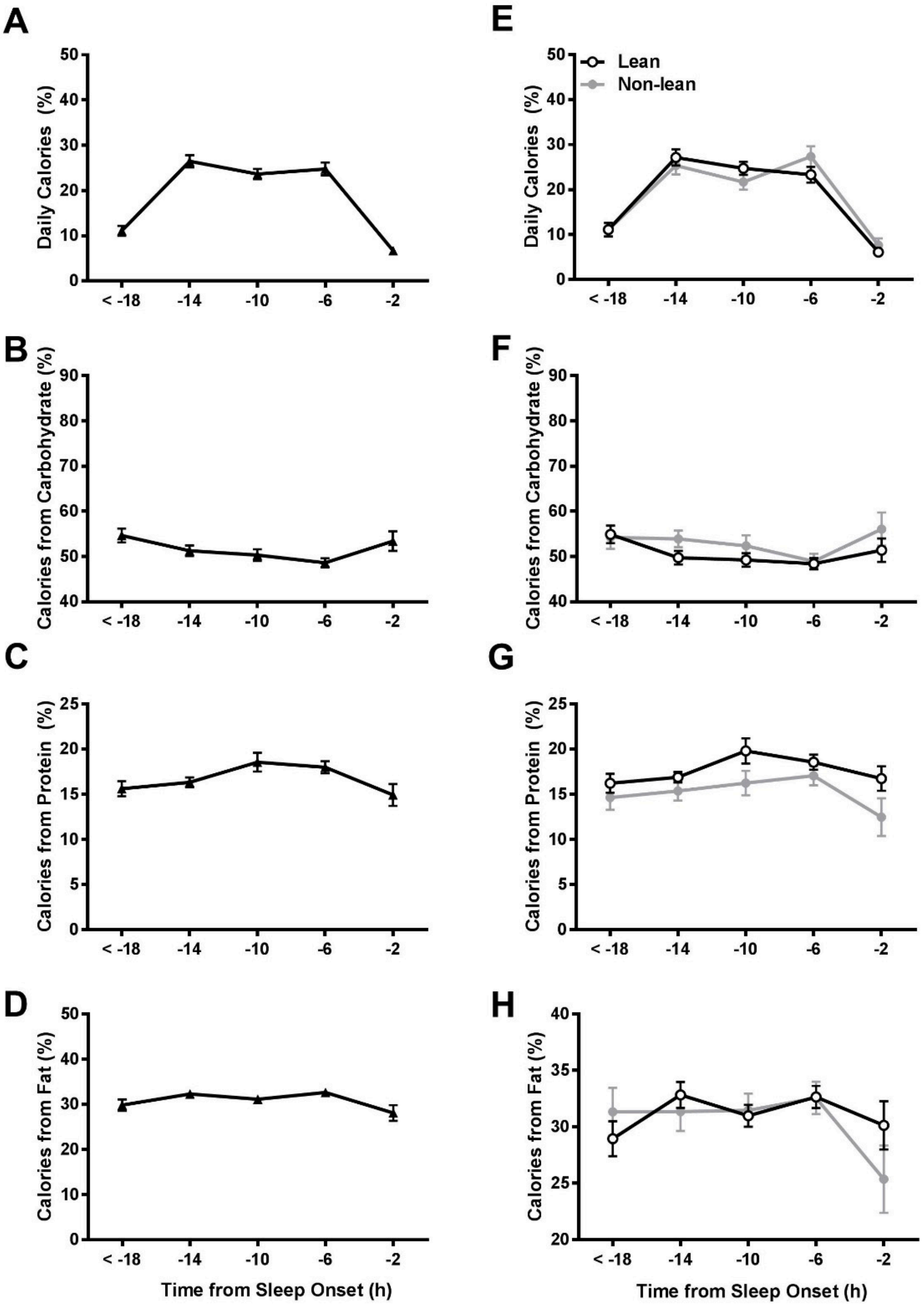


**Supplemental Figure 2.** Calories Relative to Time of Sleep Onset. Influence of time from sleep onset on (A) percent of daily calories, percent of daily calories from (B) carbohydrates, (C) proteins, (D) fat, and (E-H) percent of daily calories and each macronutrient between lean and non-lean individuals. ). All participants (n = 106) are denoted as triangles, the lean group (n = 68) is denoted by open circles and the non-lean group (n = 38) by closed gray circles. Note that the y-axis scale for daily calories from proteins (D, H) differs from the others. Error bars represent standard error of the mean.
